# Distance, rurality and the need for care: access to health services in South West England

**DOI:** 10.1186/1476-072X-3-21

**Published:** 2004-09-29

**Authors:** Hannah Jordan, Paul Roderick, David Martin, Sarah Barnett

**Affiliations:** 1 Health Care Research Unit, CCS Division, School of Medicine, University of Southampton, UK; 2 School of Geography, University of Southampton, UK; 3 International Perinatal Care Unit, Institute of Child Health, London, UK

## Abstract

**Background:**

This paper explores the geographical accessibility of health services in urban and rural areas of the South West of England, comparing two measures of geographical access and characterising the areas most remote from hospitals.

Straight-line distance and drive-time to the nearest general practice (GP) and acute hospital (DGH) were calculated for postcodes and aggregated to 1991 Census wards. The correlation between the two measures was used to identify wards where straight-line distance was not an accurate predictor of drive-time. Wards over 25 km from a DGH were classified as 'remote', and characterised in terms of rurality, deprivation, age structure and health status of the population.

**Results:**

The access measures were highly correlated (r^2^>0.93). The greatest differences were found in coastal and rural wards of the far South West. Median straight-line distance to GPs was 1 km (IQR = 0.6–2 km) and to DGHs, 12 km (IQR = 5–19 km). Deprivation and rates of premature limiting long term illness were raised in areas most distant from hospitals, but there was no evidence of higher premature mortality rates. Half of the wards remote from a DGH were not classed as rural by the Office for National Statistics. Almost a quarter of households in the wards furthest from hospitals had no car, and the proportion of households with access to two or more cars fell in the most remote areas.

**Conclusion:**

Drive-time is a more accurate measure of access for peripheral and rural areas. Geographical access to health services, especially GPs, is good, but remoteness affects both rural and urban areas: studies concentrating purely on rural areas may underestimate geographical barriers to accessing health care. A sizeable minority of households still had no car in 1991, and few had more than one car, particularly in areas very close to and very distant from hospitals. Better measures of geographical access, which integrate public and private transport availability with distance and travel time, are required if an accurate reflection of the experience those without their own transport is to be obtained.

## Background

The UK National Health Service has always aimed to provide health care for all. Although the importance of "fair access for all" (independent of the ability to pay, age, sex or area of residence) has been highlighted in recent policy documents [1], the meaning of 'fair access' is still debated [2]. Although there will always be variations in geographical access to health services, the extent of such variations and the relationship between distance to health services and the need for health care is unclear. If policy makers are to address inequities of access, more understanding is needed both of appropriate methods for measuring access and of the relationship between access to health services and health.

Although 'fair access' can be characterised simply as 'providing the right service at the right time in the right place'[3], it is a complex concept covering the provision of services, the knowledge and opportunity to use them, and the measurement of need [4]. In the UK mergers of hospital trusts have highlighted tensions between the perceived safety, effectiveness and efficiency of larger specialist centres and the demand for more geographically accessible local care [5,6], revealing the lack of evidence on which to base decisions [7]. Geographical access – the distance which must be travelled in order to use health services – is one aspect of access which is often overlooked [2], but which presents barriers of cost, time and inconvenience.

Although there is some evidence that increasing distance from health services inhibits the use of primary [8] and secondary care [9], and that it is associated with a range of poor health outcomes, from higher than expected numbers of deaths from asthma to lower than expected five year survival from cancer [10,11], few studies have attempted to quantify or set thresholds of poor access [12,13]. Furthermore, measures of geographical access can be difficult to compare. Rurality has often been used as a proxy for inaccessibility [14], as have dichotomous categorisations such as the presence or absence of a service provider in an area [15,8]. More complex measurements such as the straight line distance between populations (i.e demand points) and health service providers [16,17], or 'network distances' (which can include both road distance and travel time) [8] have added complexity, but the relationship between these measures is not clear.

One assumption which is commonly made is that geographical inaccessibility of health services is essentially a rural problem, but there is little evidence demonstrating the differences in accessibility between rural and other areas. In any area, the greatest disadvantage is likely to be experienced by individuals without access to a car (including members of one-car households without daytime access). With the declining availability of public transport, it is likely that a private car is the only convenient way to travel in rural Britain [18]. Although car ownership is relatively high in rural areas, rates for the poor, the elderly and for women are far lower than average: the 2001 Census reports that more than two thirds of single-pensioner households, many of which comprise single women, do not have access to a car. Distance may therefore be a further burden on groups with a particularly high need for health care, raising issues of inequity. Furthermore, if geographical access to health services is a problem for some groups outside of traditional rural areas, then rural policies alone will not tackle the problem.

In this paper, we aim to determine the geographical accessibility of health services, and the demographic and health related factors associated with it. We compare two measures of geographical access: straight-line distance and modelled drive-time along the road network to primary and secondary care throughout South West England. We investigate whether the most geographically inaccessible populations are in rural areas, describe the relationship between geographical access to hospital and population health, and investigate whether the populations furthest from health services have a greater need for health care due to age or deprivation.

The study area is the former South West Region, comprising the counties of Avon, Cornwall and the Isles of Scilly, Devon, Dorset, Gloucestershire, Hampshire, the Isle of Wight, Somerset and Wiltshire. As defined in 1991 this area has a population of about 6 million, with a low proportion from ethnic minorities, and a higher than average proportion living in rural areas.

## Results

### Correlation between the access measures

**Figure 1:**
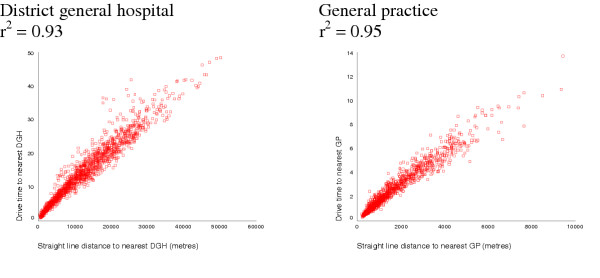
Correlation between straight line and drive-time measures to GP and hospital services

**Figure 2:**
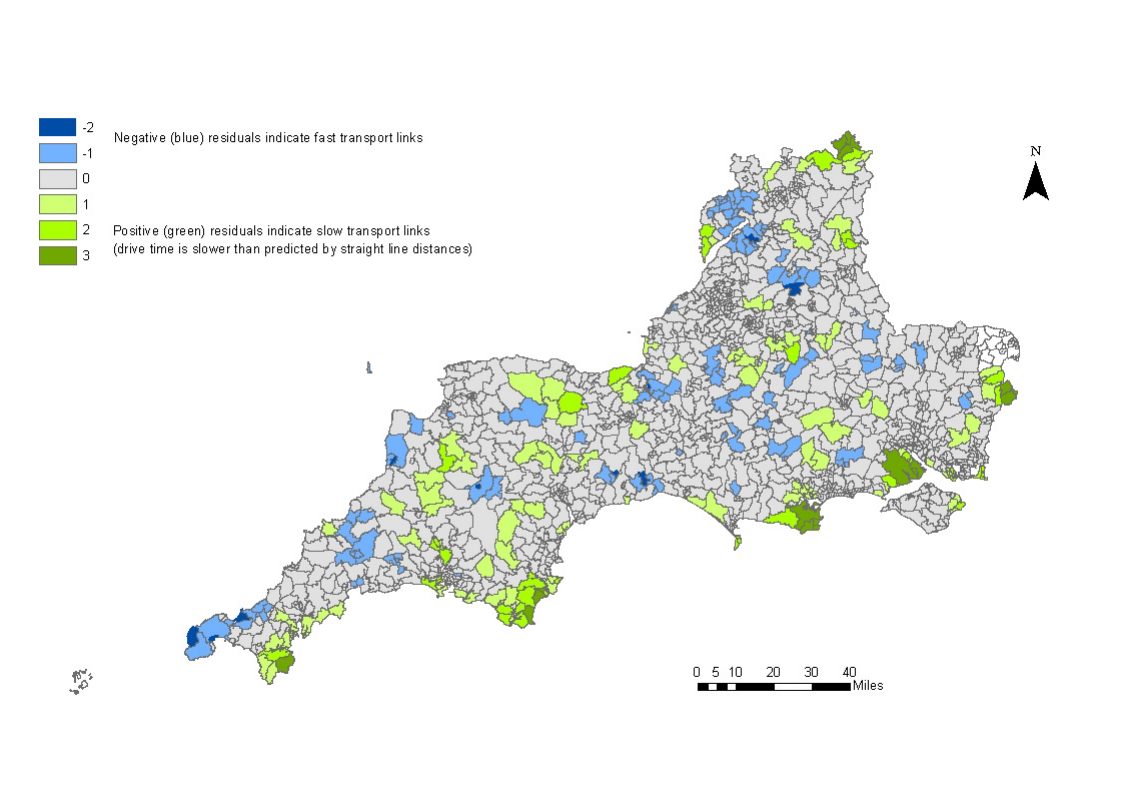
Standardised residuals from the regression of drive time and straight-line distance to hospitals

The straight-line and drive-time measures were highly correlated for both GP and hospital services (figure 1). Areas where residuals from the regression analysis of straight-line distance and drive-time to DGHs are more than two standard deviations from the norm were concentrated in coastal and rural wards of the far South West. Areas where residuals are negative indicate faster than expected drive times, wheras positive residuals indicate that drive time is slower than predicted by straight line distances (figure 2). The analysis was repeated, excluding wards along the boundary between the study area and neighbouring counties to check for edge-effects, but there was no difference in results.

### Distances to health services

Distances to GPs were low, with a median distance of just 1 km to the closest practice (IQR 0.6 – 2.2). The calculation was repeated excluding branch surgeries (which tend to have limited opening hours), but this made little difference to the outcome, with a median distance to a main surgery of just 1.2 km. 95% of wards (98% of the population) were under 4.4 km, or 6.3 minutes, from their closest GP. The maximum distance to a GP was just 9.4 km (13.7 minutes). The median distance to a DGH was just less than 12 km (IQR 5.4 – 19.0), with a maximum of 50 km, corresponding to an estimated 13 and 48 minutes drive-time (table 1).

**Table 1:** Access to DGHs and GPs

		**25^th ^centile**	Popn (%)*	**Median**	**Popn (%)**	**75^th ^centile**	**Popn (%)**	**95^th ^centile**	**Popn (%)**	**Maximum**
**Straight line (km)**	**DGH**	5.4	2.40 (39.3)	11.6	3.97 (65.1)	19.0	5.15 (84.3)	29.0	5.92 (97.1)	50.1
	**GP surgery**	0.6	2.24 (36.8)	1.0	4.17 (68.3)	2.2	5.39 (88.4)	4.4	5.96 (97.7)	9.4
**Drive time ('minutes')**	**DGH**	7.1	2.38 (38.9)	13.4	3.93 (64.4)	20.5	5.17 (84.7)	31.6	5.93 (97.2)	48.3
	**GP surgery**	1.0	2.19 (35.9)	1.7	4.00 (65.5)	3.4	5.28 (86.5)	6.3	5.89 (96.5)	13.7

*Population in millions (percent of the total population) living in wards within this distance of their closest DGH and GP

### Remoteness and rurality

For the purposes of this study, remoteness from health services was defined as over 5 km from a GP or over 25 km from a DGH. Access to primary care was good, with just 91 wards (6.3% of the total) remote from primary care. These areas have just 3% of the regional population. Of these the majority (63%) were ONS 'rural' areas. There were 162 wards which we classified as remote from hospitals (11% of the total, home to 6.5% of the region's population). All had drive-times to hospital of over 21 minutes; 81 (51%) were urban by the ONS classification, 69 (43%) were rural areas and the remaining eight (5%) were rural fringe. Four wards had no ONS urban / rural classification (table 2).

**Table 2:** ONS rurality and remoteness from hospital

**N (%)**	**Rural**	**Rural fringe**	**Not rural**	**No classification**	Total*
**Remote**	69 (43%)	8 (5%)	81 (51%)	2 (1%)	162 (100%)
**Not remote**	184 (14%)	146 (11%)	950 (74%)	6 (0.5%)	1286 (100%)

### Distance and the need for health care

**Figure 3:**
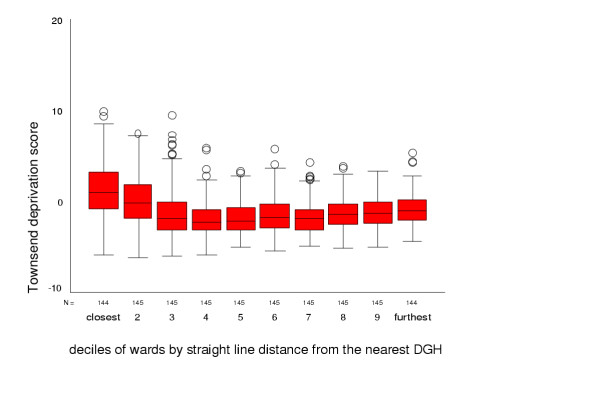
Townsend deprivation score for deciles of wards by straight-line distance from DGH

Deprivation scores ranged from -6.2 to 9.9, with a mean of -1.0, indicating that the study area had a slightly more affluent profile than the England and Wales average. The most affluent wards were in the middle of the range of straight-line distances from secondary care. The median deprivation score was 1.0 in the decile of wards closest to hospitals, decreased to a low of -2.2 in the 5^th ^decile, then rose steadily to -1.0 in the decile of wards furthest from hospitals, giving a slight 'U' shape to the relationship between deprivation and distance from health services (figure 3).

**Figure 4:**
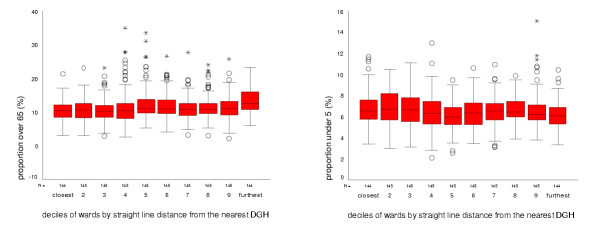
**Age structure of wards by straight-line distance from DGH. **average proportion of young (under 5) and elderly (over 65) population for deciles of Wards by straight line distance from DGH

The proportion of over 65 year olds increased slightly with straight-line distance from hospitals: more remote wards had a slightly higher proportion of residents over the age of 65, but there was considerable variation within deciles of remoteness, and the observed difference was small. The proportion of the population under five years old in 1991 showed no clear trend with ward distance from hospital, but was slightly lower in more remote wards (figure 4).

The age-standardised rate of LLTI was highest in the areas closest to hospitals. The LLTI rate decreased with increasing distance from hospital and then increased again in the most remote areas. Standardised rates of premature mortality showed no strong pattern with distance from a hospital, although median rates were high in areas close to hospitals and also slightly raised in the most remote areas. The proportion of households with no car was highest in the areas closest to hospital, but increased again in the decile of wards furthest from hospital. The same pattern was seen in the ownership of two or more cars – the lowest rates were found in areas either very close to or very far from hospitals (table 3).

**Table 3:** Median values for health outcomes and car ownership for deciles of ward by straight-line distance from DGH

	**Closest**	**2**	**3**	**4**	**5**	**6**	**7**	**8**	**9**	**Furthest**
**LLTI SMR (0–64)**	1.08	1.02	0.92	0.84	0.85	0.89	0.85	0.87	0.89	1.02
**All-cause SMR (0–64)**	1.08	0.99	0.93	0.91	0.87	0.93	0.88	0.91	0.96	0.94
**Proportion of households with No car**	34.2	29.3	25.4	20.4	20.3	21.0	20.1	20.7	20.2	23.1
**Two or more cars**	20.0	23.2	27.8	32.1	33.2	32.0	32.8	32.5	31.2	27.0

## Discussion

The impact of distance on the use of hospitals and other health care, and on health status, has not been well established. In the UK threshold distances of between 24 and 50 miles to specialist hospital services [19,20], 10 miles to screening services [21], 7 km (4 miles) to family planning clinics [22] and 2.5 miles to primary care [23] have all been used in reporting 'poor access', but there is little consensus and no strong theoretical or empirical basis for these choices. By international standards, distances to health services for our study population are low, averaging just 12 km to the closest hospital, but drive-times to hospital of up to 50 minutes are predicted by our model and there are groups who could be considerably disadvantaged by the travel distances we have reported.

A variety of measures of geographic access of varying complexity and specificity exist and selecting an appropriate measure is not simple. Straight-line distances are widely used, easy to calculate and to compare and, in this study, they are closely correlated with the more complex drive-times. However, there is some evidence that areas of low correlation are concentrated in peripheral areas of the rural South West. In these areas straight-line distances underestimate true travel distance, reflecting sparse road networks and geographical barriers such as hills, rivers and coastline. Access to health services in these areas could be misrepresented by the use of the simpler measure, masking problems faced by these populations. Furthermore, neither measure used here reflects the experience of those without access to a private car. Travel to hospital and GP appointments is already known to be a problem for some groups in rural areas of the UK. Although informal systems of 'lift-giving' and more formal 'voluntary taxi' schemes often exist [24] these are not available everywhere [25,26], and it could be argued that a measure of travel by public transport is vital in determining accessibility for the most disadvantaged populations. Few studies have attempted this [27-29]., and composite measures, which include both public and private transport, are even less common [30]. Better measures of access, which integrate private and public transport, are required to reflect the experience of those on low incomes, and without their own transport.

A surprising finding of this study was the relatively low proportion of wards remote from health care which are defined as 'rural'. Fewer than half of the wards remote from hospital and under two-thirds of those remote from primary care are classified as rural by the ONS. Analysis which concentrates on rural areas under the ONS definition, or even stretches this to include 'rural fringe' areas, will still miss over half of the wards which are remote from hospitals. There has been concern over the targeting of resources in concentrations of deprivation: the majority of deprived people live outside of these areas and are not reached by narrowly focused initiatives. Although the ward-level definition of rurality used here may class as 'urban' some small towns which many would consider essentially 'rural' when viewed at a larger scale (such as the Local Authority level), we conclude that caution should be exercised when evaluating and responding to poor access to health services, a high proportion of which occurs outside areas traditionally considered to be remote.

In this study, we found no clear threshold at which need becomes greater or health status sharply declines. If anything, the converse is true with worse health status and greatest need in areas close to health services. Distance to health care was not associated with a high proportion of elderly or very young residents, but was related to deprivation. We found high deprivation in areas close to hospitals, relative affluence in more distant areas and an increase in deprivation in the most remote wards. Deprivation indices have been criticised for failing to represent deprivation in rural areas [31] and the relatively high proportion of rural areas in the most remote wards may hide even higher need in these areas. Further research using different measures of need and deprivation is indicated.

Although the highest rates of morbidity and mortality were found in the areas closest to hospitals, there was some evidence of increasing rates in more remote areas. Rates of LLTI, particularly for those under 64, show an upwards trend in more remote areas. This supports previous findings that LLTI is higher in rural wards with the most dispersed populations [31], but it is not clear whether this reflects a true increase in morbidity or a perception of handicap of those living in such areas. The relationship between distance and all-cause premature mortality is less clear. The high levels of mobility which are often reported in populations living far from services were upheld by our study (expressed through high car ownership), but the areas most remote from hospitals begin to show a decrease in levels of car ownership. It is unlikely that this indicates less need for private transport, and may indicate a less wealthy population for whom travel is a potential problem.

There are a number of important limitations to our study. We have explored only one region of England, a relatively affluent area with a very small ethnic minority population and an unusual 'peninsular' geography. Our findings need to be reproduced in other areas. We have limited our definition of access to simple geographical measures. Other aspects of accessibility include the quantity and quality of health services, and financial and cultural barriers to their use, and have not been explored here. The choice of SMRs and LLTI rates as health outcome indicators may have resulted in our inability to observe stronger relationships between geographical access and health: even over a six year period absolute numbers of deaths were low. More research is needed including the young and elderly and using a wider range of health status measures. Finally, the inter relationship between use of health care, need and access has been insufficiently explored.

## Conclusions

This paper has provided a population-based estimate for access to both primary and secondary health care in South West England. We have shown that although geographical access to health services is generally good, remoteness from health services is an issue which affects both urban and rural areas. Studies concentrating purely on rural areas are therefore likely to underestimate the extent of geographical barriers to accessing health care. Areas which were furthest from hospitals did not have an especially old or young population, but there was some evidence of higher rates of LLTI and of deprivation in the most remote wards, indicating higher need for services in the areas furthest from them. Finally, the fact that almost a quarter of households in the decile of wards most remote from hospital services had no car in 1991 indicated a large number of people for whom travel is likely to be more difficult than implied by current measures of geographical access.

Our understanding of the effect of distance on the use of services and on health outcomes is far from complete. Both the measurement of access and the understanding of need and deprivation require further exploration. The development of web-based public transport information systems may supply the data needed to enhance currently available measures of access by adding public transport travel times, likely to be relevant to access for the poorest and most deprived populations and the introduction of the Indices of Multiple Deprivation 2000 in England may present a clearer picture of the need for health care than traditional census-based indices [32]. This index contains a measure of geographical access to services, which has been of particular interest to rural populations and may provide a missing dimension to the measurement of deprivation. Linking geographical access with a wider range of health status measures and health care use in different populations is also vital if a clear picture of the impact of accessibility of health care is to be fully understood.

## Methods

### Measuring geographical access to health services

A Geographical Information System (Arc/Info) and custom written programs were used to calculate two measures of access to health services. Access was calculated from all residential postcodes to primary care services (all main and branch General Practice (GP) surgeries) and secondary care services (acute hospitals (DGHs)) in the region. Data on main and branch GP surgeries (n = 1469) were obtained from all Family Health Services Authorities in 1998. Acute DGHs (n = 39) were defined as hospitals with general medicine and general surgery facilities and an Accident and Emergency department. DGHs were identified using the hospital year-books (1992–97) and hospitals were contacted to clarify their status in 1997 as necessary.

The first access measure calculated was a widely used measure: the shortest straight-line distance between every residential postcode, the closest GP (both main and branch) and the closest DGH. A more complex measure of access was the shortest drive-time from each residential postcode to the closest GP and the closest DGH. This was modelled using estimated road-network travel speeds along the Bartholomew digital road network and associating these with residential postcode locations by the use of a travel time (drive-time) surface model. While including provision for congestion and slow travel through urban areas, this measure does not include any estimates for parking times or transfers between car and surgery or hospital. The methods are described in detail elsewhere [33].

### The need for health care

Proxy measures of the need for health care were calculated. The Townsend score, a widely used indicator of material deprivation, was calculated from 1991 census data. The variables used in the score are the percentage of economically active people over the age of 16 who are unemployed; the percentage of households which are overcrowded; the percentage of households with no car and the percentage of households not owning their own home. A log transformation is applied to the overcrowding and unemployment variables. The logged variables and the car ownership and owner occupation variables are standardised by creating z-scores for each value, and the four z-scores are summed to provide the final Townsend score. Scores are standardised to give a mean of zero for England and Wales: any scores greater than zero indicate relative deprivation, any less than zero represent relative affluence. The proportions of the population over 65 and under 5 years old – were taken from the 1991 Census Small Area Statistics (SAS). Health status was assessed using indirectly standardised rates of all-cause mortality and Limiting Long Term Illness (LLTI) for all those under 65 (premature mortality and morbidity). Data on LLTI were taken from the 1991 Census. The Office for National Statistics (ONS) provided data on all-cause mortality for the years 1991–1996. Data were aggregated over the six years, to minimise problems due to small numbers of cases in some wards.

### Assigning data to geographical areas

Postcodes were allocated to 1991 Census wards using the 1991 and subsequent postcode to enumeration district directories. Travel times and distances were calculated for all residential postcodes and aggregated to ward level for analysis. The resident population of each ward was used to weight individual postcode times and distances to create a population-weighted average, as demonstrated in table 4. ONS ward classifications were used to select 'rural' wards [34]. The ONS classifications are listed in table 5. Only two categories: 'rural areas' and 'rural fringe', are unambiguously rural under this definition and we have defined than as rural here. All other wards were defined as urban.

**Table 4:** Aggregating household level access data to wards

**Ward**	**Postcode**	**N Households from each postcode in Ward1**	**Time from PC to health services**	Households * time
Ward1	PC1	10	10	100
Ward1	PC2	7	13	91
Ward1	PC3	2	11	22
Ward1	PC4	6	21	126
Sum (Ward1)		25		339

Population weighted average time for Ward1 ((hhds*time)/hhs)

**Table 5:** The ONS ward classification

ONS group	Rural / urban classification
Suburbia	Urban
Rural areas	Rural
Rural fringe	Rural
Industrial areas	Urban
Middling Britain	Urban
Prosperous areas	Urban
Inner city estates	Urban
Established owner occupiers	Urban
Transient populations	Urban
Metropolitan professionals	Urban
Deprived city areas	Urban
Lower status owner occupiers	Urban
Mature populations	Urban
Deprived industrial areas	Urban

### Analyses

To investigate if straight-line distance was a valid proxy for the more complex drive-time measure, the two were compared using Pearson correlation coefficients and a regression analysis of drive-time against straight-line distance. Areas where straight-line distance appeared to underestimate the drive-time more than expected were identified and mapped to investigate the extent of geographical clustering. Access to primary and secondary health services was described using median distances and inter-quartile ranges for both measures.

To investigate the assumption that it is the residents of rural areas who are most disadvantaged by poor geographical access to health services, we first had to define poor access. Standard estimates of 'remoteness' from health services have not been established – there is no a priori definition of the distance regarded as 'remote from health services' and no consensus has been established in the literature on access to health services. The proportion of rural, rural fringe and urban wards which were 'remote' from health services under the definition of a straight-line distance of three, five or seven kilometres to a GP and 20, 25, 30 or 35 km to a hospital was therefore calculated (Table 6). We used an arbitrary cut-off point of a straight-line distance of 5 km to a GP and 25 km to a hospital, beyond which wards were classed as 'remote' from health services. These distances classified approximately 6% of the study population as remote from secondary care and 3% as remote from primary care.

**Table 6:** ONS rurality and remoteness from primary and secondary care

	**Rural**	**Rural fringe**	**Urban**	**No classification**	**Total wards**
**All wards**	253 (18%)	154 (11%)	1031 (71%)	10 (1%)	1448 (100%)
**GPs**					
**Remote (3 km)**	117 (53%)	14 (6%)	84 (38%)	6 (3%)	221 (100%)
**Remote (5 km)**	20 (53%)	4 (10%)	12 (32%)	2 (5%)	38 (100%)
**Remote (7 km)**	5 (71%)	1 (14%)	0 (0%)	1 (14%)	7 (100%)
**Hospitals**					
**Remote (20 km)**	126 (39%)	36 (11%)	158 (49%)	4 (1%)	324 (100%)
**Remote (25 km)**	69 (43%)	8 (5%)	81 (51%)	2 (1%)	162 (100%)
**Remote (30 km)**	30 (49%)	1 (2%)	28 (46%)	2 (3%)	61 (100%)
**Remote (35 km)**	17 (59%)	0 (0%)	12 (41%)	0 (0%)	29 (100%)

We then identified the proportion of remote wards that were rural under the ONS classification. To investigate relationships between distance to health services and the need for health care, straight-line distance to hospital was used to group wards into deciles and the deprivation score and the age profile of the population in each decile was described. Standardised rates for premature all-cause mortality and LLTI were used to indicate health outcomes for each decile of wards, and car ownership (as reported in the 1991 census) was used to indicate how easy travel would be for the population in each group.

## Authors' contributions

HJ carried out the analyses and drafted the manuscript. HJ, PR, and DM collaborated in the formation of the research questions, the management of the study and the development of the paper. HJ and SB collected the data and calculated measures of deprivation, health and accessibility. DM designed the drive-time access measure. All authors read and approved the final manuscript.
